# In Situ SEM-EBSD Tensile Study of GH4169 Alloy with Different Grain Sizes

**DOI:** 10.3390/ma19163411

**Published:** 2026-08-11

**Authors:** Jinyuan Yang, Wenqiang Zhang, Shuang Chen, Fangfeiyuan Zhang, Jiayi Tian, Jin Wang

**Affiliations:** School of Materials Science and Engineering, Zhejiang University, Hangzhou 310058, China; 22326025@zju.edu.cn (J.Y.); 12326083@zju.edu.cn (W.Z.); chen_shuang@zju.edu.cn (S.C.); 22326070@zju.edu.cn (F.Z.); 12626006@zju.edu.cn (J.T.)

**Keywords:** in situ, grain size, microstructure, crystal orientation, Schmid factor

## Abstract

**Highlights:**

- The influence of precipitated phases was eliminated via heat treatment process control, ensuring that the grain size served as the sole variable.
- In situ SEM dynamically tracks microstructural morphology evolution of specimens during tensile testing.
- The grain size and Schmid factor jointly affect the tensile behavior of materials.

**Abstract:**

To explore the effect of grain size on the tensile deformation mechanism of GH4169 nickel-based superalloy, specimens with average grain sizes of 10.7 μm and 70.6 μm were fabricated via different heat treatment processes, and in situ tensile experiments were carried out at room temperature using combined in situ SEM-EBSD technology. The results show that the fine-grained specimen exhibits a yield strength of 794 MPa and an ultimate tensile strength of 1372 MPa, while the coarse-grained specimen presents a yield strength of 317 MPa and an ultimate tensile strength of 782 MPa. During tensile loading, the fine-grained specimen undergoes uniform deformation with strong grain boundary coordination, accompanied by homogeneous grain orientation rotation; its average kernel average misorientation (KAM) value increases moderately from 0.82 to 0.89. In contrast, the coarse-grained specimen suffers severe inhomogeneous deformation and readily generates continuous slip bands, featuring abrupt local changes in grain orientation, with the KAM value rising from 0.8 to 1.04. Slip systems with high Schmid factors induce localized intragranular deformation. Benefiting from a high grain boundary density, the fine-grained specimen effectively restrains strain localization and achieves more uniform bulk deformation of the alloy.

## 1. Introduction

Owing to its excellent high-temperature strength, corrosion resistance and good processability, GH4169 alloy is widely used in the manufacturing of high-end equipment such as aerospace and nuclear power industry [1,2,3,4]. During actual service, components made of this alloy sustain tensile loads for the long term [5,6], and their tensile deformation behavior directly determines the load-bearing capacity and service safety of the components [7,8,9]. Therefore, a systematic study on the tensile behavior and its influencing mechanism of GH4169 alloy is of great theoretical value and practical significance for ensuring the reliable operation of equipment and promoting the upgrading of its engineering applications [10,11]. Grain size is one of the key microstructural factors affecting the tensile properties of GH4169 alloy [12,13,14]. GH4169 alloy contains various precipitates, mainly including γ″-phase, γ′-phase and δ-phase [15,16,17]. Thulasiram et al. conducted tensile tests on GH4169 alloy with different heat treatments. Their study indicated that a decreased grain size contributed to a decreased cleavage size, which leads to increased ductility [18]. In existing studies investigating the tensile and fatigue mechanical behavior of this alloy, the δ-phase and grain size are usually comprehensively analyzed as combined influencing factors. However, it should be noted that the δ-phase exhibits distinctly different action mechanisms on the mechanical properties of GH4169 alloy with varying grain sizes [19,20,21], which interferes with the independent investigation of the grain size effect. At present, most studies on the tensile mechanical behavior of GH4169 alloy adopt ex situ observation methods [22]. That is, the tensile deformation mechanism is indirectly analyzed by combining the initial microstructural morphology and tensile fracture morphology of the alloy. However, the post-fracture microstructure can only reflect the final state of the tensile process and cannot accurately characterize the dynamic influence of various factors during tensile deformation [23,24,25]. Meanwhile, this method fails to capture the real-time evolution of the microstructure during tensile deformation, making it difficult to effectively track the key influencing factors of the tensile mechanical properties of GH4169 alloy, resulting in an insufficiently in-depth understanding of its deformation mechanism.

To address the shortcomings in the above-mentioned studies, differential heat treatment was applied to GH4169 alloy in this investigation. By precisely controlling the heat treatment parameters, the interference of the δ-phase on tensile properties was effectively eliminated, and two types of specimens with different grain sizes were successfully prepared. On this basis, in situ scanning electron microscopy (in situ SEM) was employed to track the dynamic evolution of the surface microstructure of the specimens in real time. Combined with in situ electron backscatter diffraction (EBSD) characterization data, the evolution laws of the Schmid factor and crystal orientation during tensile deformation of specimens with different grain sizes were emphatically analyzed. This work provides experimental support for further revealing the tensile deformation mechanism of GH4169 alloy.

## 2. Materials and Methods

### 2.1. Experimental Material

In this study, the typical age-hardened nickel-based superalloy GH4169 (international designation: Inconel 718) was selected as the experimental material, and the base metal was a commercial alloy produced by NCS Testing Technology Co., Ltd. (Beijing, China). To eliminate the interference of precipitates on the tensile properties of GH4169 alloy, two different heat treatment processes were adopted. The specific parameters of the two heat treatment processes are as follows: Heat treatment process A: Heated uniformly to 980 °C at a heating rate of 30 °C/min, held at the predetermined temperature for 1 h, and then immediately cooled by water quenching. Heat treatment process B: Heated uniformly to 1050 °C at a heating rate of 30 °C/min, held at the predetermined temperature for 1 h, and then cooled by water quenching as well. After the above two different heat treatments, fine-grained and coarse-grained GH4169 alloy specimens can be obtained, respectively. There is a significant difference in grain size between the two types of specimens, which meets the regulation requirements of the core variables in this study. To obtain a surface suitable for electron backscatter diffraction (EBSD) characterization, the specimens were ground with silicon carbide (SiC) abrasive papers from 240# to 5000#. Electropolishing was then performed in an electrolyte with a volume ratio of HClO_4_:C_2_H_5_OH = 1:9. The process was carried out at 25 °C with a constant voltage of 18 V for 15 s.

### 2.2. In Situ Tensile Testing

**Figure 1:**
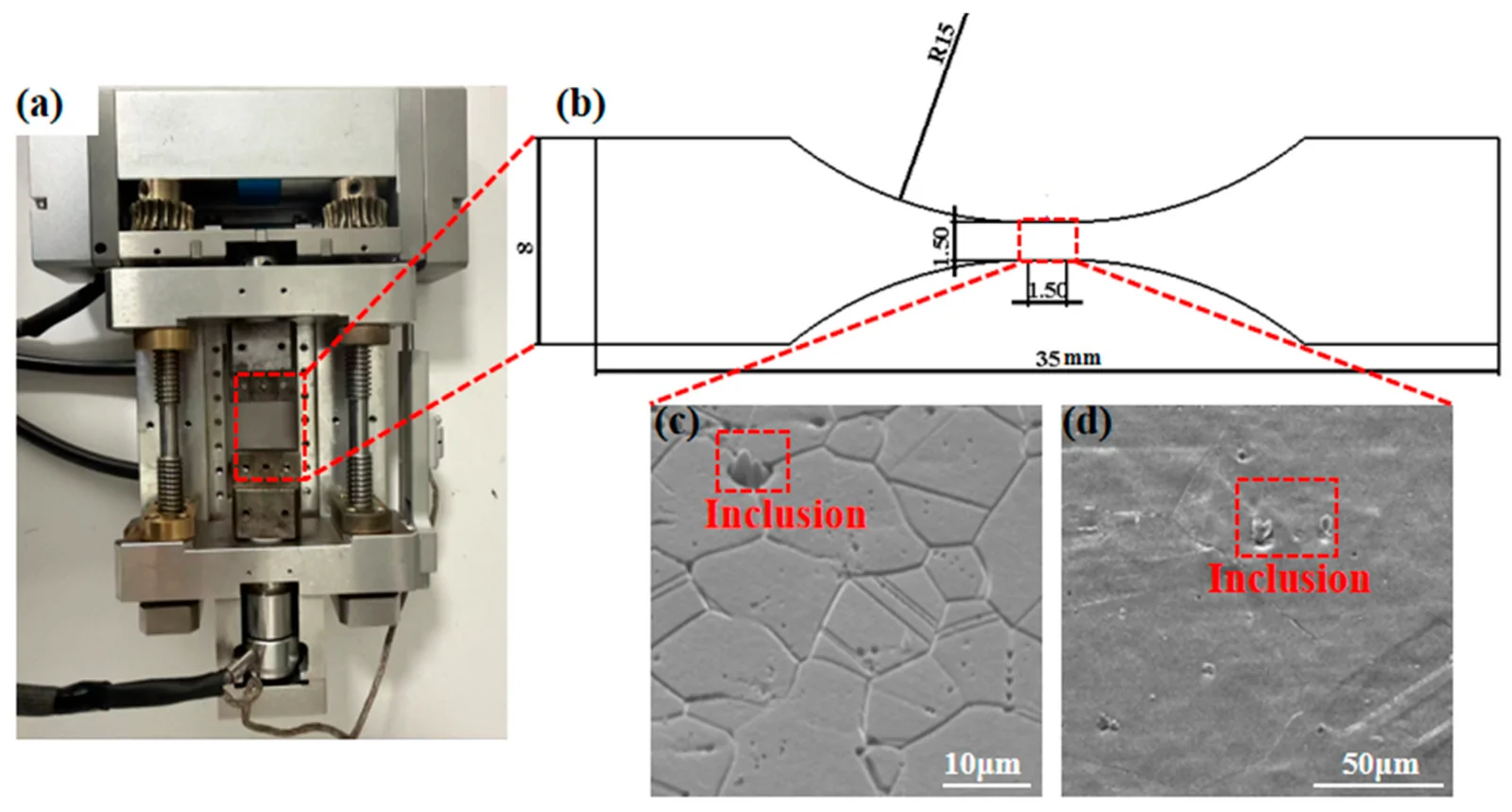
(**a**) In situ tensile stage diagram; (**b**) specimen diagram; (**c**,**d**) initial SEM morphology images of fine-grained and coarse-grained specimens.

The in situ tensile tests were carried out at room temperature using an in situ tensile stage compatible with a scanning electron microscope (Tescan 8000, produced by TESCAN GROUP, a. s., Brno, Czech Republic) chamber, as shown in Figure 1a. In situ tensile tests were performed separately on fine-grained and coarse-grained GH4169 alloy specimens. The observed region in the tests is marked in red in Figure 1. During the experiment, the tensile rate was fixed at 1 μm/s. The thickness of both specimens is 1 mm. In this experiment, the specimen was tilted at 70°. The SEM accelerating voltage was set to 20 kV, the electron beam current was 1 nA, and the working distance was 20 mm. The EBSD mapping area for the fine-grained specimen is 175 × 130 μm, while that for the coarse-grained specimen is 600 × 450 μm. In general, the EBSD scanning step size is set to less than 1/10 of the average grain size. To further improve the accuracy of grain identification, a step size of 0.3 μm was adopted for the fine-grained specimens. Given the larger grain size of coarse-grained specimens, a correspondingly expanded EBSD scanning area is required. To prevent specimen drift caused by an excessively long data acquisition time, the step size for coarse-grained specimens was set to 1 μm. The grain boundary misorientation threshold angle is set to 10°, and twin boundaries are identified based on a 60° rotation about the <111> axis. The initial indexing rates were 99.65% and 99.68% for fine-grained and coarse-grained specimens, respectively. The EBSD indexing rate gradually decreased with increasing deformation. When the displacement exceeded a certain value, the EBSD quality deteriorated sharply, rendering the data unavailable. To investigate the evolution of surface microstructure and crystallographic orientation of the alloy at different deformation extents, surface micrographs of both groups of specimens were captured, and electron backscatter diffraction (EBSD) data were synchronously collected when the displacement was 0 μm, 600 μm and 900 μm, respectively. Figure 1c,d show the initial SEM micrographs of the fine-grained and coarse-grained specimens, respectively. SEM observations indicate that no δ-phase precipitation is detected in the two specimens, which is in good agreement with the expected treatment outcomes. A small amount of inclusions can be found within both specimens. As internal defects in the alloy, inclusions affect the distribution and transmission of internal stress [26,27], and thereby alter the mechanical performance of the specimens during the subsequent tensile tests [28].

## 3. Results

### 3.1. Initial EBSD Information

**Figure 2:**
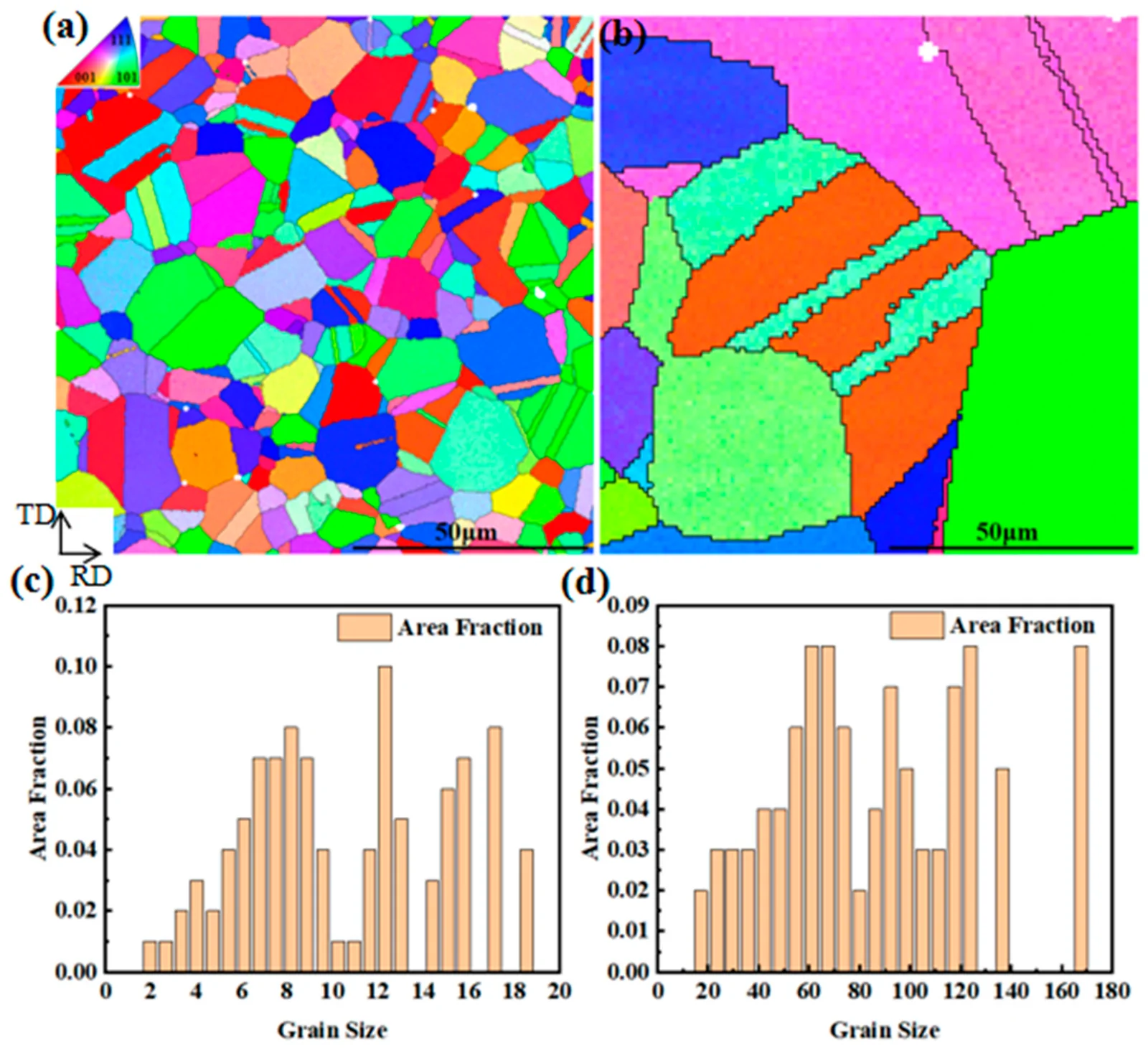
(**a**) Initial EBSD map of the fine-grained specimen; (**b**) initial EBSD map of the coarse-grained specimen; (**c**) grain size distribution chart of the fine-grained specimen; (**d**) grain size distribution chart of the coarse-grained specimen.

Figure 2a,b show the initial EBSD maps of the fine-grained and coarse-grained specimens, respectively. The specific processing procedures are as follows: Low-confidence pixels were eliminated via CI filtering, and isolated pixels were removed. A single pass of 3 × 3 Kuwahara smoothing was applied to deformed specimens to prevent over-smoothing, while two passes were carried out for undeformed specimens. According to the EBSD analysis, annealing twins are formed in both specimens, which are generated during the heat treatment processes mentioned above. As an important component of the alloy microstructure, the formation of twins changes the crystal orientation distribution inside the alloy [29,30], thereby affecting the mechanical properties, such as plastic deformability and strength of the alloy [31]. Specifically, GH4169 possesses an FCC austenitic crystal structure, and the twin-matrix pair exhibits a misorientation angle of 60°, which increases the orientation diversity of the specimen. RD refers to the loading direction, and TD is perpendicular to the loading direction. The raw EBSD data collected above were systematically processed and analyzed using AZtecCrystal 2.1. The equivalent circle diameter (ECD) was used to characterize the grain size. The statistical results of the grain size for the two specimens are shown in Figure 2c,d. The specimen subjected to heat treatment A (980 °C for 1 h + water quenching) has a relatively small grain size, with an average grain size of 10.7 μm (fine-grained specimen). In contrast, the specimen treated by heat treatment B (1050 °C for 1 h + water quenching) exhibits clear grain growth due to the higher holding temperature, and its average grain size reaches 70.6 μm (coarse-grained specimen). A significant difference in the average grain size is observed between the two specimens.

**Figure 3:**
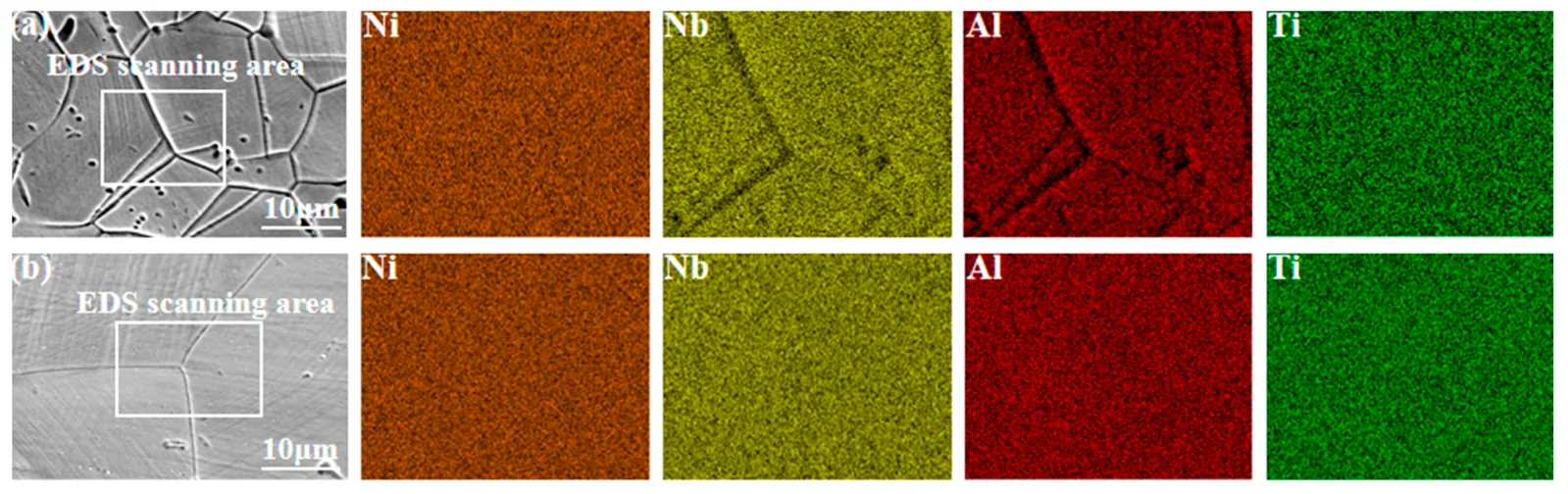
(**a**) EDS mapping of the fine-grained specimen; (**b**) EDS mapping of the coarse-grained specimen.

EDS mapping was performed on fine-grained and coarse-grained specimens. The results reveal a homogeneous element distribution without local segregation in both samples, demonstrating that no clear precipitates are generated after heat treatment. Accordingly, grain size is confirmed as the sole variable, as presented in Figure 3.

### 3.2. Tensile Curves

**Figure 4:**
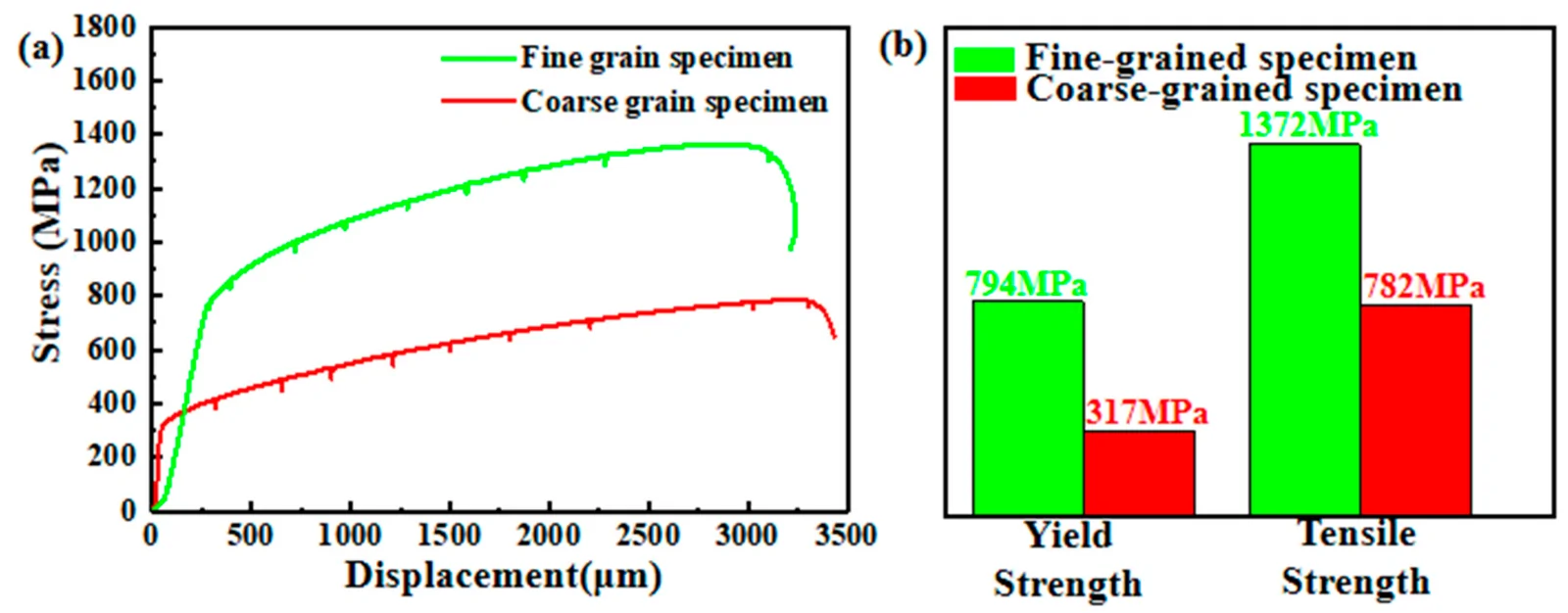
(**a**) Tensile stress–displacement curve; (**b**) comparison of mechanical properties.

The in situ tensile stress–displacement curves of the fine-grained and coarse-grained specimens are shown in Figure 4a. The displacement is measured by the built-in grating ruler of the in situ system, which represents the total deformation of the specimen excluding the gauge section. The fine-grained specimen has a yield strength of 794 MPa and an ultimate tensile strength of 1372 MPa, while the coarse-grained specimen exhibits a yield strength of 317 MPa and an ultimate tensile strength of 782 MPa, as shown in Figure 3b. It should be noted that during the in situ tensile tests, the experiment was paused intermittently to clearly capture the real-time evolution of the surface microstructure of the specimens. During this pause, due to the suspension of the in situ tensile stage, the tensile mechanical curve shows a characteristic vertical drop. This is a normal phenomenon in the experiment and is not caused by specimen damage or experimental errors. EBSD data of both fine-grained and coarse-grained specimens were synchronously acquired at the two critical deformation stages with displacements of 600 μm and 900 μm. Limited by equipment conditions and test duration, the present in situ characterization tests mainly aim to reveal the underlying microscopic mechanisms.

### 3.3. Microstructural Evolution

**Figure 5:**
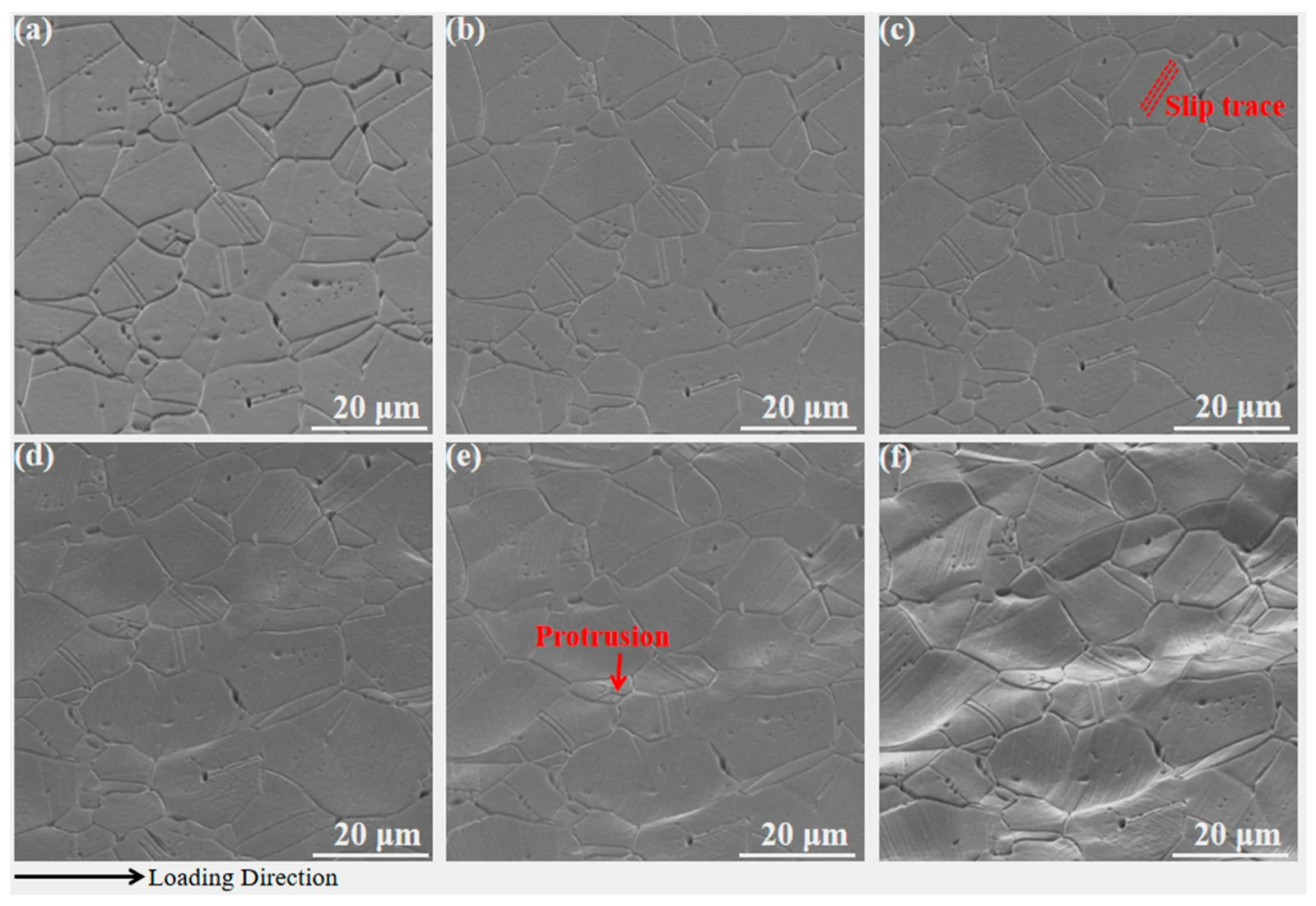
Surface morphology of fine-grained specimen: (**a**) ∆L = 0 μm; (**b**) elastic stage; (**c**) yield stage; (**d**) ∆L = 600 μm; (**e**) ∆L = 900 μm; (**f**) ∆L = 1800 μm.

Figure 5 shows sequential SEM images illustrating the surface microstructural evolution of the fine-grained specimen with increasing tensile strain, which clearly presents the microstructural characteristics and deformation behavior of the specimen at different deformation stages. As shown in Figure 5a, when the specimen is in the initial undeformed state, the surface is flat and smooth with a small number of inclusions, and no δ-phases are observed. The grains exhibit a uniform and fine equiaxed distribution, indicating that the microstructure of the specimen after heat treatment is homogeneous. When the specimen is in the elastic deformation stage, the applied load is mainly supported by the interatomic bonding forces within the alloy. The atoms only experience elastic displacement without any plastic slip [32]. Therefore, no clear microstructural changes appear on the specimen surface, which remains generally flat, as shown in Figure 5b. After the specimen enters the yielding stage, the applied load exceeds the yield strength of the alloy, the interatomic bonding forces are broken, and plastic deformation initiates in the material. Fine and short slip traces emerge within multiple grains. At this stage, intragranular slip acts as the dominant deformation mode. The occurrence of slip traces indicates the activation of dislocation motion inside the grains [33], as presented in Figure 5c. When the displacement reaches 600 μm, the specimen enters the stage of uniform plastic deformation. As the deformation continues to increase, dislocation motion is intensified and the number of slip traces rises remarkably. Several parallel slip bands form inside some grains, and some slip traces even extend across the entire grain, indicating an expanded slip range. Meanwhile, slight coordinated deformation appears at grain boundaries, which is caused by the small relative displacement of adjacent grains to accommodate the overall plastic deformation, as shown in Figure 5d. When ∆L = 900 μm, plastic deformation is further enhanced. Slip bands continuously propagate and intersect with each other, forming fine slip networks in some grains, indicating the interaction and entanglement of dislocations, which increases the slip resistance. Slight surface undulation occurs due to local inhomogeneous deformation and aggravated grain-boundary slip, as illustrated in Figure 5e. When the displacement reaches 1800 μm, clear undulations appear on the specimen surface, and the inhomogeneity of deformation is further aggravated. Coordinated deformation at grain boundaries becomes more significant, and distinct grain-boundary sliding traces can be seen. Some grain boundaries even show a slight separation trend, suggesting that grain-boundary sliding has become an auxiliary deformation mechanism, which cooperates with intragranular slip to accommodate plastic deformation, as displayed in Figure 5f.

**Figure 6:**
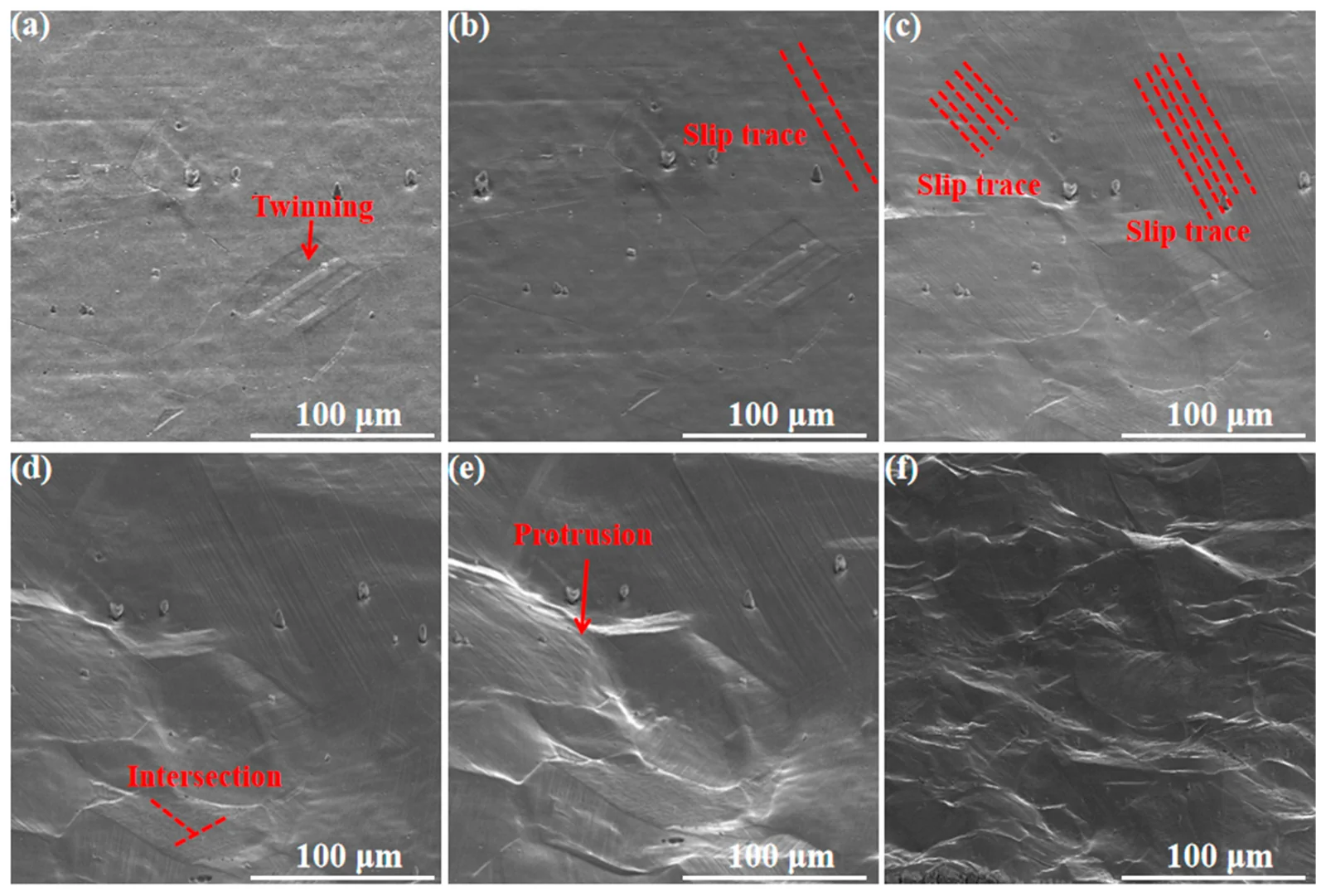
Surface morphology of coarse-grained specimen: (**a**) ∆L = 0 μm; (**b**) yield stage; (**c**) ∆L = 600 μm; (**d**) ∆L = 900 μm; (**e**) ∆L = 1800 μm; (**f**) ∆L = 2200 μm.

Figure 6 shows sequential SEM images of the surface microstructural evolution of the coarse-grained specimen during in situ tensile testing. In the initial state, the coarse-grained specimen has a smooth surface with a small number of inclusions. Clear twin bands can be observed after polishing and etching, as shown in Figure 6a. When the specimen enters the yielding stage, sparse and slender shallow slip traces emerge on the surface of partial grains, whereas no clear morphological variation can be observed in the remaining regions, as illustrated in Figure 6b. When the displacement reaches 600 μm, the specimen enters the uniform plastic deformation stage. Dislocations proliferate drastically inside the grains, and distinct slip traces can be observed on the surface of most grains. The slip bands continuously widen and elongate, gradually forming continuous slip bands traversing the entire grains, as presented in Figure 6c. When the displacement reaches 900 μm, the slip bands further develop and intersect with one another to form distinct slip steps, and prominent protrusions can be observed on the specimen surface, as shown in Figure 6d. When the displacement reaches 1800 μm, drastic undulations emerge on the specimen surface and remarkable plastic flow occurs within the grains, as shown in Figure 6e. When the displacement increases to 2200 μm, the specimen presents an overall wavy macroscopic undulation and even undergoes necking, as illustrated in Figure 6f. In situ SEM results reveal that the grain size directly determines the maximum slip length of dislocations. Fine grains possess smaller slip plane dimensions, and the average dislocation slip distance is much shorter than that in coarse grains. The short slip path restricts the propagation of slip bands within individual grains, preventing localized concentrated deformation induced by coarse slip bands. Consequently, plastic deformation is distributed across more grains, leading to more uniform deformation.

## 4. Discussion

### 4.1. Changes in Crystal Orientation

**Figure 7:**
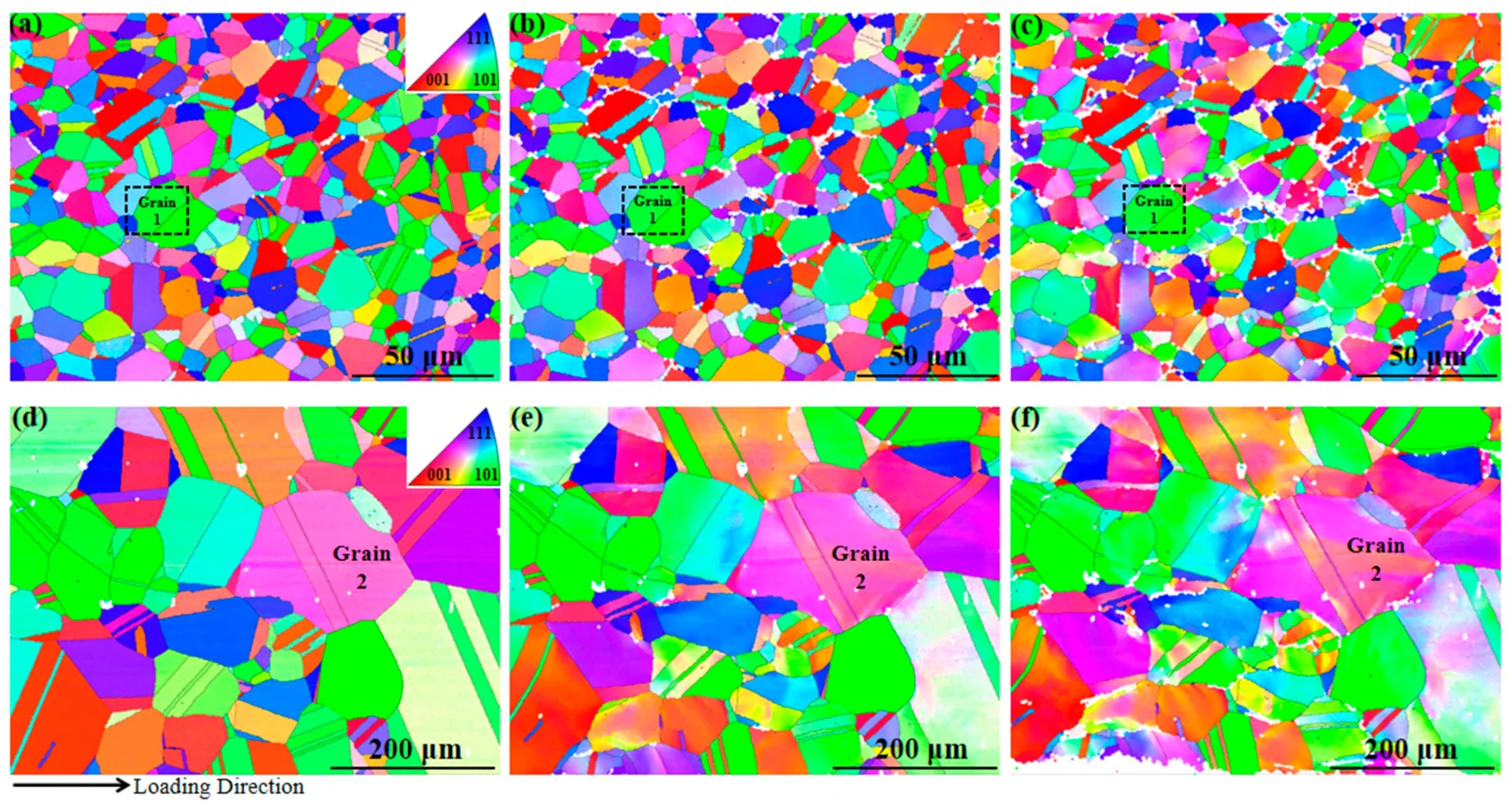
IPF comparison chart: (**a**) fine-grained specimen ∆L = 0 μm; (**b**) fine-grained specimen ∆L = 600 μm; (**c**) fine-grained specimen ∆L = 900 μm; (**d**) coarse-grained specimen ∆L = 0 μm; (**e**) coarse-grained specimen ∆L = 600 μm; (**f**) coarse-grained specimen ∆L = 900 μm.

Figure 7 shows the grain orientation evolution of fine-grained and coarse-grained specimens under different displacement, further revealing the effect of grain size on the microdeformation mechanism of GH4169 alloy. Previous studies using ex situ characterization methods have confirmed that the overall grain orientation of GH4169 alloy gradually converges to the [111] orientation during tensile deformation [34], which is consistent with the results shown in Figure 7 of this work. Combined with in situ EBSD observations, it is further found that grain size significantly affects the orientation evolution behavior of the alloy during tension. For fine-grained specimens, grains undergo continuous and uniform dispersed rotation under tensile loading, with almost no local abrupt changes in orientation (Figure 7b,c). In contrast, coarse-grained specimens exhibit weakened grain boundary constraint. Deformation tends to initiate preferentially and concentrate locally within grains with high Schmid factors. Only a small number of grains experience clear preferential rotation, and local large-angle misorientation accumulates continuously with increasing strain (Figure 7e,f).

**Figure 8:**
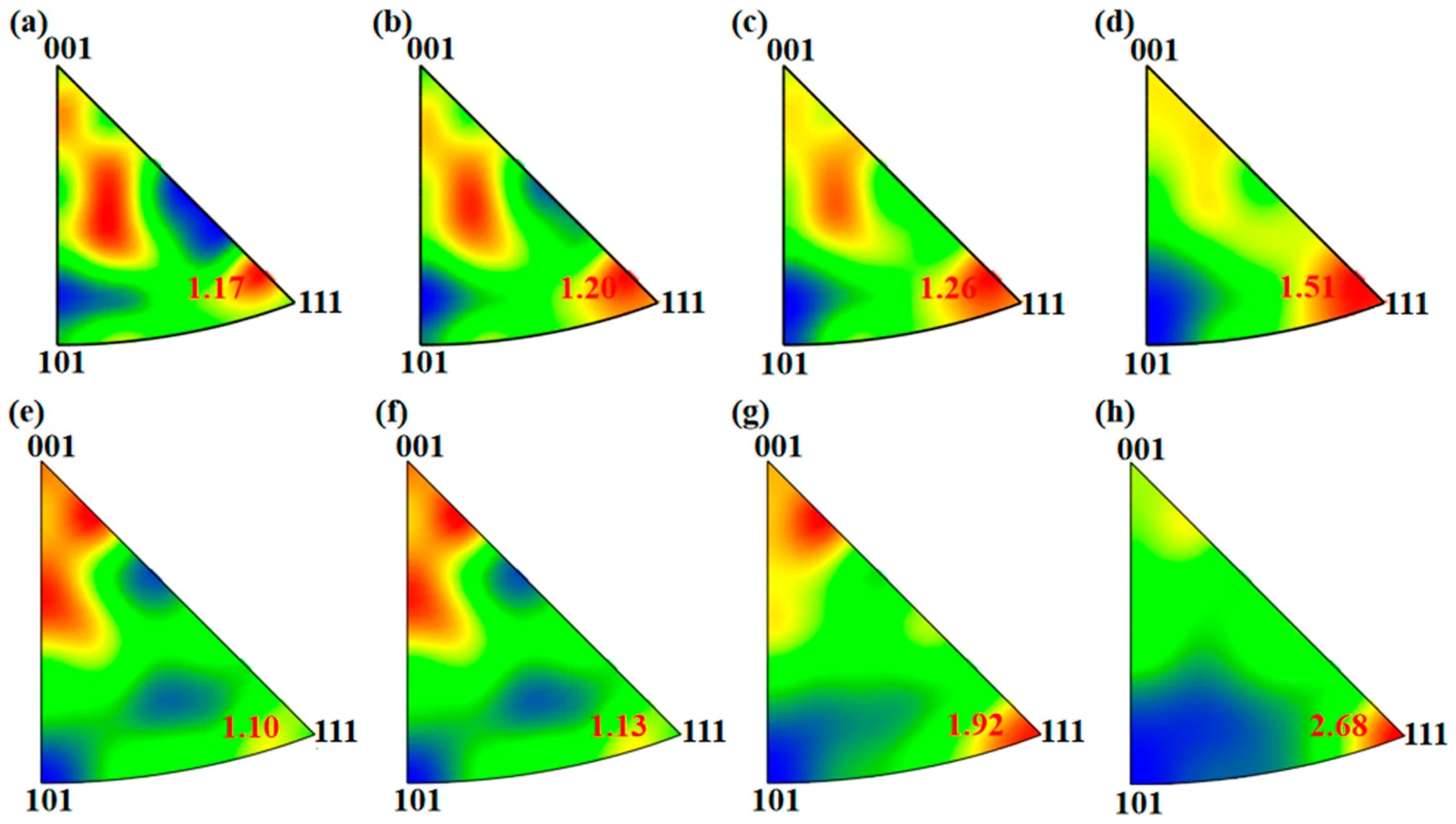
Inverse pole figures of specimens under different strains: (**a**) fine-grained specimen ∆L = 0 μm; (**b**) fine-grained specimen at yield; (**c**) fine-grained specimen ∆L = 600 μm; (**d**) fine-grained specimen ∆L = 900 μm; (**e**) coarse-grained specimen ∆L = 0 μm; (**f**) coarse-grained specimen at yield; (**g**) coarse-grained specimen ∆L = 600 μm; (**h**) coarse-grained specimen ∆L = 900 μm.

Furthermore, Figure 8 shows the inverse pole figures (IPFs) of fine-grained and coarse-grained specimens under different tensile strains, which reflect the evolution of crystal texture during deformation. Existing studies have generally observed that [111] texture readily forms in GH4169 alloy after tensile deformation, yet the dynamic evolution of texture with strain remains poorly understood [35]. Experimental results indicate that when the displacement is 900 μm, the texture intensity of the fine-grained specimen slowly increases from the initial value of 1.17 to 1.51 and remains at a relatively low level. In contrast, the texture intensity of coarse-grained specimens increases sharply from 1.10 to 2.68, forming a strong [111] preferred orientation. The underlying mechanism for such differences is as follows. Fine-grained microstructures feature a high grain boundary density, and strong intergranular constraints activate multiple slip systems cooperatively, leading to mutual restriction of grain orientation rotation. For coarse-grained microstructures, grain boundaries provide limited constraint. Deformation is concentrated on dominant slip systems, which induces long-range preferential rotation of grains and ultimately results in significant texture enhancement.

**Figure 9:**
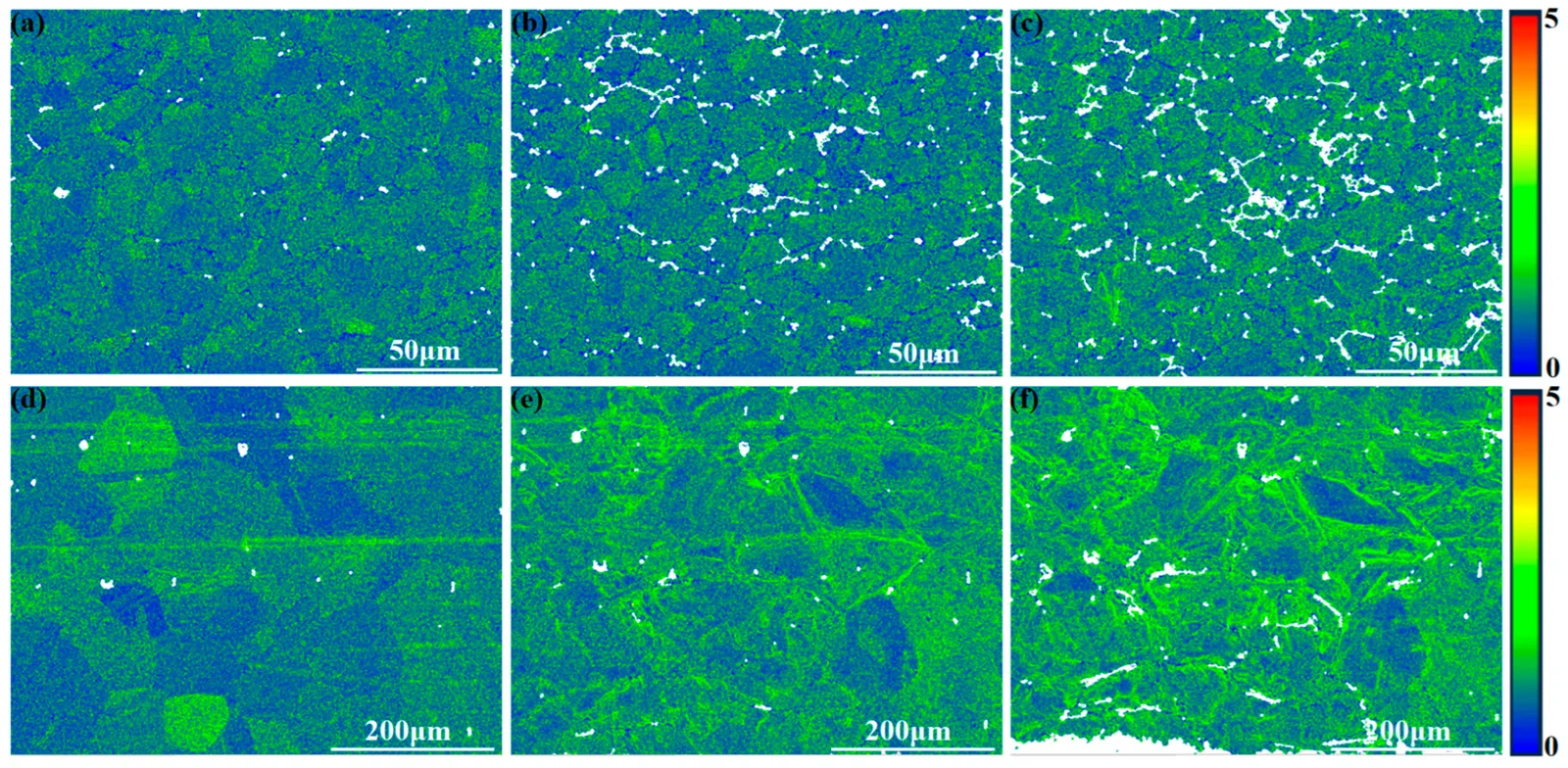
KAM comparison chart: (**a**) fine-grained specimen ∆L = 0 μm; (**b**) fine-grained specimen ∆L = 600 μm; (**c**) fine-grained specimen ∆L = 900 μm; (**d**) coarse-grained specimen ∆L = 0 μm; (**e**) coarse-grained specimen ∆L = 600 μm; (**f**) coarse-grained specimen ∆L = 900 μm.

Previous ex situ tests have revealed that under tensile loading, strain tends to concentrate at slip bands and grain boundaries in coarse-grained GH4169 alloy specimens [36], whereas the strain field distributes uniformly in fine-grained ones. To clarify the intrinsic mechanism behind this difference, in situ observation was adopted to dynamically analyze the KAM distribution of the two specimens at various deformation stages, as presented in Figure 9. Considering the large overall deformation of the specimens, a threshold angle of 5° was applied for KAM calculation [37]. The neighborhood order of KAM is set to the first order. The fine-grained specimens maintain low and uniformly distributed KAM values throughout the tensile process. Grain boundaries act as “strain barriers”, which effectively disperse local stress and inhibit strain concentration, as shown in Figure 9a–c. In contrast, the high-KAM regions in coarse-grained specimens rapidly expand and interconnect with increasing strain. Stress propagates and accumulates along long-range slip bands, resulting in severe strain localization, as displayed in Figure 9d–f.

**Figure 10:**
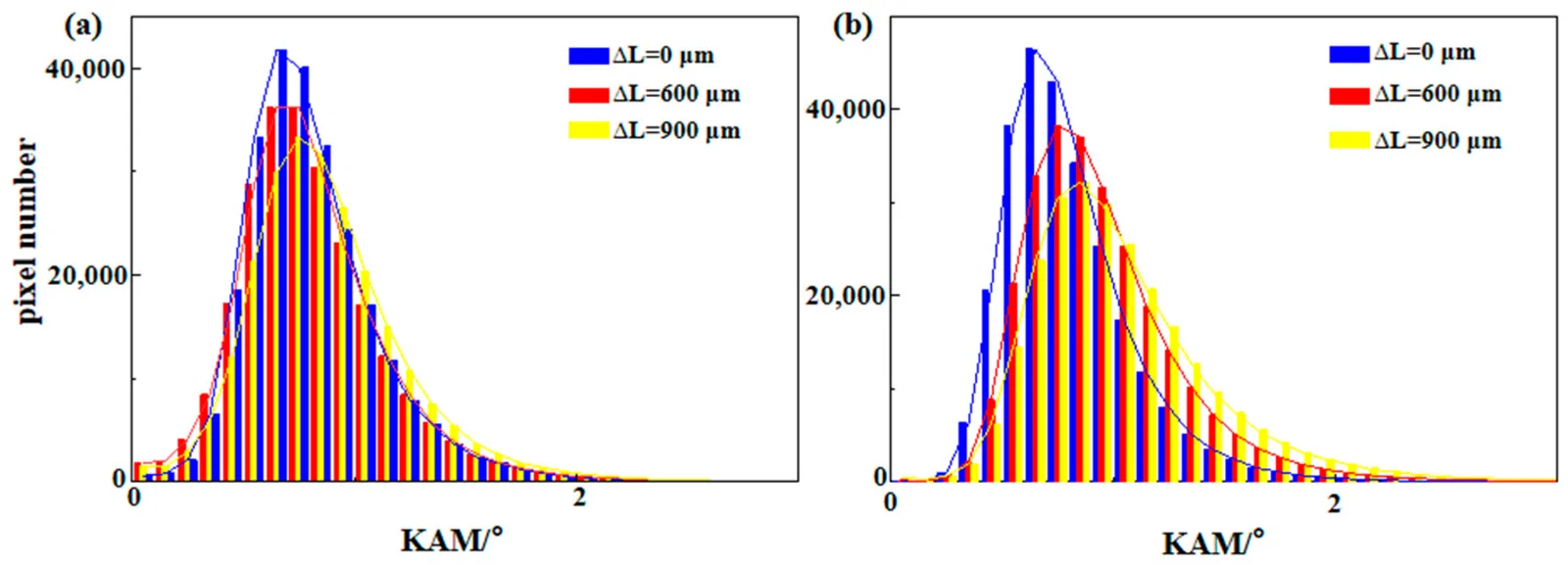
KAM histogram: (**a**) fine-grained specimen; (**b**) coarse-grained specimen.

Figure 10 presents the histograms of KAM distribution under different displacement conditions. Since the proportion of data with KAM values exceeding 3° is extremely low, such data are excluded for a clearer presentation. For the fine-grained specimen, the average KAM value is 0.82, the median value of KAM is 0.75 at ∆L = 0 μm, and the average KAM value barely rises at ∆L = 600 μm. The average KAM value reaches 0.89, and the median value of KAM is 0.85 at ∆L = 900 μm, which is consistent with the uniform deformation characteristic of fine-grained materials. For the coarse-grained specimen, the average KAM value is 0.80, and the median value of KAM is 0.75 at ∆L = 0 μm. The average KAM value increases to 0.95, and the median value of KAM increases to 0.85 at ∆L = 600 μm. The average KAM value rises to 1.04, and the median value of KAM is 0.95 at ∆L = 900 μm. This further verifies the inhomogeneous deformation of coarse grains.

### 4.2. Schmid Factor

**Figure 11:**
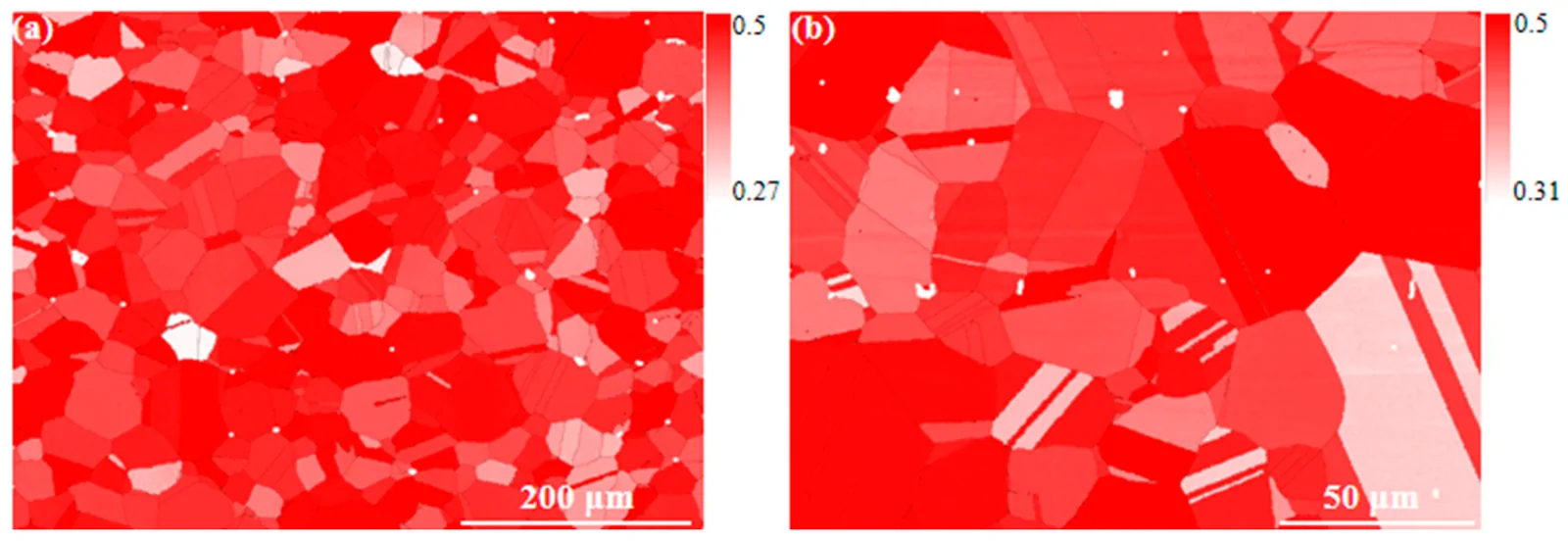
Schmid factor maps: (**a**) fine-grained specimen; (**b**) coarse-grained specimen.

During tensile deformation, the deformation behavior of the specimen is related not only to crystal orientation but also to the Schmid factor of grains. As a key parameter quantifying the difficulty of slip system activation with respect to crystal orientation, the Schmid factor further refines the regulatory effect of crystal orientation on deformation behavior. The Schmid factor ranges from 0 to 0.5. A higher value indicates a more favorable orientation relationship between the slip system and the tensile loading direction, a lower critical resolved shear stress required for slip system activation, and thus easier activation of the slip system to participate in plastic deformation. Conversely, a lower Schmid factor makes slip system activation more difficult. Grains then undergo plastic slip less readily and tend to bear external load via grain boundary accommodation. Figure 11 presents the Schmid factor distribution maps of the two specimens. It can be seen that there is no clear correlation between grain size and the Schmid factor of individual grains. However, the significant misorientation between twins and the matrix leads to distinct Schmid factors.

**Figure 12:**
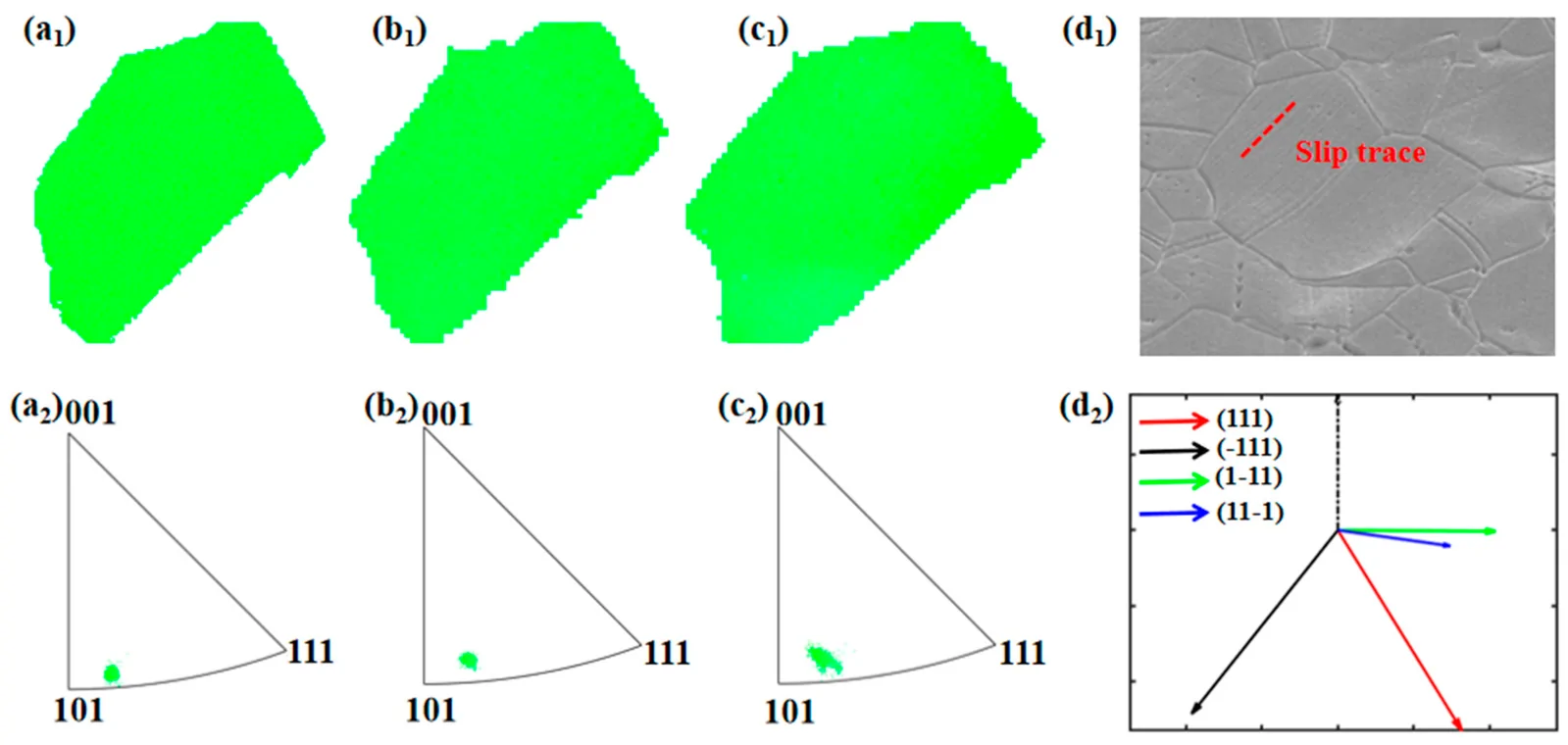
Orientation evolution of Grain1 in the fine-grained specimen across the loading direction: (**a_1_**,**a_2_**) ∆L = 0 μm; (**b_1_**,**b_2_**) ∆L = 600 μm; (**c_1_**,**c_2_**) ∆L = 900 μm; (**d_1_**) surface slip traces; (**d_2_**) theoretical surface slip trace directions of each slip system.

The Mtex 5.11.1 toolbox in MATLAB 2019 was used to analyze the typical grains of both specimens. Figure 12 shows the orientation evolution of the selected typical grain Grain1 in the fine-grained specimen along the loading direction. Slip trace analysis indicates that partial slip traces within this grain are activated preferentially. Calculation results confirm that the initially activated slip system corresponds to the slip system with a higher Schmid factor in Grain1. Figure 12(a_2_), 12(b_2_) and 12(c_2_) present the orientation distribution of Grain1 at ∆L = 0 μm, ∆L = 600 μm and ∆L = 900 μm, respectively. With tensile deformation proceeding, the grain is elongated significantly. Its orientation at different strains scatters almost randomly. Inhomogeneous intragranular plastic deformation occurs in the grain during tension. The calculated Schmid factors of this grain are listed in Table 1. According to Table 1, the (-111)<110> slip system of Grain1 exhibits relatively high Schmid factors. A higher Schmid factor indicates that the corresponding slip system is more easily activated during tensile deformation. Therefore, the above slip system is activated preferentially and dominate the plastic deformation of the grain, resulting in inhomogeneous plastic deformation within the grain.

**Figure 13:**
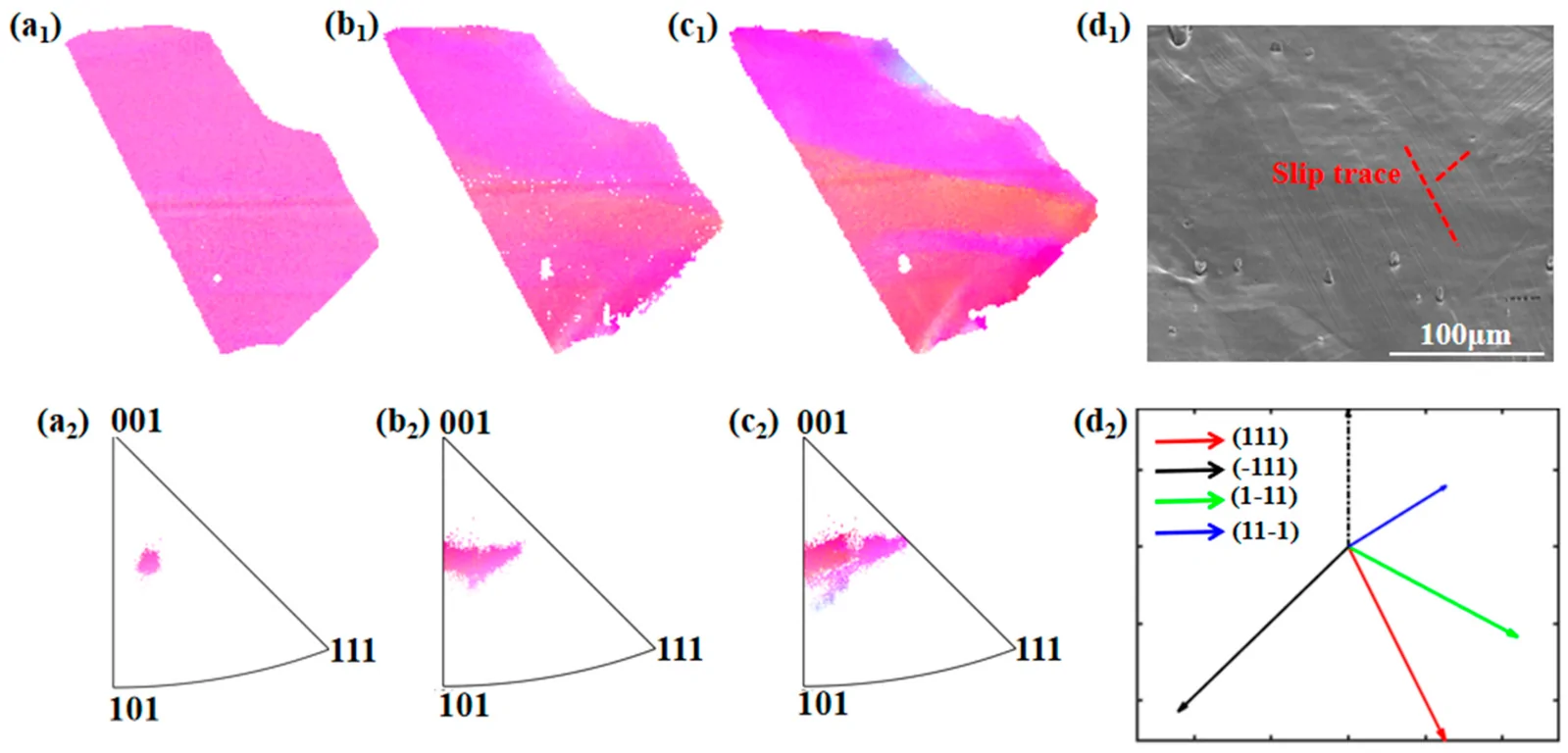
Orientation evolution of Grain2 in the coarse-grained specimen along the loading direction: (**a_1_**,**a_2_**) ∆L = 0 μm; (**b_1_**,**b_2_**) ∆L = 600 μm; (**c_1_**,**c_2_**) ∆L = 900 μm; (**d_1_**) surface slip traces; (**d_2_**) theoretical surface slip trace directions of each slip system.

Figure 13 illustrates the orientation evolution of a typical grain in the coarse-grained specimen during tensile deformation. Slip trace analysis reveals that some slip traces in this grain initiate first. Calculations verify that the preferentially activated slip system is the one with a relatively large Schmid factor in Grain2. After being loaded, the grain elongates along the loading direction, with no clear tendency toward a specific orientation. The Schmid factor of Grain2 was calculated and is listed in Table 2. Similar to Grain1, Grain2 has two easily activated slip systems: the (111)<-101> and (-111)<101> slip systems. This indicates that when Grain2 enters the plastic deformation stage, the (111)<-101> and (-111)<101> slip systems with higher Schmid factors will be activated preferentially.

Compared with Grain2, Grain1 exhibits a less scattered orientation and a lower degree of inhomogeneous plastic deformation inside the grain. At the single-grain scale, slip systems with high Schmid factors are preferentially activated in both fine and coarse grains, resulting in inhomogeneous intragranular deformation and concentrated slip bands. In grains with low Schmid factors, slip is difficult to initiate, and the intragranular deformation is relatively uniform. At the polycrystalline scale, the grain size significantly weakens or amplifies the preferential effect of Schmid factors by regulating the coordination capacity of grain boundaries. The grain boundary densities of the two specimens were calculated. The fine-grained specimen has a grain boundary density of 0.3027, while that of the coarse-grained specimen is 0.0466. The fine-grained specimen possesses abundant grain boundaries and strong constraints between adjacent grains. Even if some grains with high Schmid factors tend to deform preferentially, such deformation can be rapidly dispersed and offset by surrounding grains via grain boundary coordination. This weakens the influence of Schmid factors on the overall deformation and achieves more uniform deformation of the alloy. In contrast, the coarse-grained specimen has fewer grain boundaries and weaker intergranular constraints. Once deformation initiates in grains with high Schmid factors, the surrounding grains fail to provide effective coordination. Deformation remains concentrated within a small number of grains, eventually leading to severe strain localization, penetration of slip bands, intensified texture and deteriorated plasticity.

## 5. Conclusions

In this study, the grain size of GH4169 alloy was regulated by heat treatment. In situ SEM was used to real-time observe the changes in microstructure during the tensile process of the specimens, and in situ EBSD was combined to analyze the crystal orientation rotation and the influence of the initial grain Schmid factor of the two specimens during the tensile process.

1. In situ EBSD observations reveal that crystal orientations in both fine- and coarse-grained specimens rotate toward the [111] direction under tension. Coarse-grained specimens show stronger texture evolution, while fine-grained specimens exhibit weak texture development. Due to the high grain boundary density, fine grains can effectively activate multiple slip systems and disperse the tendency of grain orientation rotation, so the textural strength remains in the weak range. In contrast, coarse grains have insufficient grain boundary constraints, deformation is dominated by a few dominant slip systems, and grains undergo unconstrained long-range preferential rotation.
2. If a single grain has slip systems with high Schmid factors, plastic deformation will be concentrated on such slip systems, leading to inhomogeneous intragranular deformation; if the Schmid factors of all slip systems are relatively low, deformation will be dispersed among multiple slip systems, resulting in more homogeneous intragranular deformation.
3. The grain boundary density of the fine-grained specimen reaches 0.3027, which endows adjacent grains with a strong capacity for coordinated deformation. Even if partial grains possess high Schmid factors and tend to deform preferentially, the synergistic effect of numerous surrounding grains rapidly disperses local strain and alleviates strain localization. In contrast, the coarse-grained specimen only has a grain boundary density of 0.0466. Once plastic slip initiates preferentially in grains with high Schmid factors, the surrounding grains cannot effectively share the applied load, resulting in continuous strain accumulation within a small number of grains and consequently inhomogeneous deformation.

## Figures and Tables

**Table 1:** Grain1 Schmid factor.

(111)			(-111)			(1-11)			(11-1)		
[0-11]	[-101]	[-110]	[0-11]	[101]	[110]	[011]	[-101]	[110]	[011]	[101]	[-110]
0.0323	0.3705	0.4028	0.0295	0.4075	0.4371	0.0046	0.0020	0.0026	0.0665	0.0349	0.0316

**Table 2:** Grain2 Schmid factor.

(111)			(-111)			(1-11)			(11-1)		
[0-11]	[-101]	[-110]	[0-11]	[101]	[110]	[011]	[-101]	[110]	[011]	[101]	[-110]
0.3065	0.4658	0.1593	0.2599	0.4992	0.2392	0.3399	0.2117	0.1282	0.2264	0.1782	0.0482

## Data Availability

The original contributions presented in this study are included in the article. Further inquiries can be directed to the corresponding author.
